# Neurotransmitter‐related functional connectivity alterations associated with cognitive decline in people with mild cognitive impairment and mild Alzheimer’s disease

**DOI:** 10.1002/alz.095384

**Published:** 2025-01-09

**Authors:** Annalena Venneri, Riccardo Manca

**Affiliations:** ^1^ Brunel University London, London United Kingdom; ^2^ University of Parma, Parma Italy; ^3^ Brunel University London, Uxbridge United Kingdom

## Abstract

**Background:**

Early alterations in the ventral tegmental area, a major brainstem dopaminergic nucleus, may be a marker of Alzheimer’ disease (AD). However, how dopamine (DA) may influence neurofunctional and cognitive decline due to AD remains elusive. The aim of this study was to investigate alterations in resting‐state functional connectivity (FC) associated with DA distribution in the brain along the AD continuum.

**Materials:**

Eighty‐six older adults with mild cognitive impairment (MCI), 58 with mild dementia due to AD and 76 cognitively unimpaired (CU) participants of the multicentre VPH‐DARE@IT project were included. Participants underwent comprehensive clinical, neuropsychological and MRI assessments

**Methods:**

Functional MRI data were pre‐processed by using the REACT pipeline to obtain FC maps informed by DA receptor and transporter PET/SPECT atlas for the whole brain and for the nigrostriatal and mesocorticolimbic pathways. Whole‐brain FC maps informed by acetylcholine (ACh) were also extracted for comparison. A cognitive composite score (Cog‐CS) was calculated as the average of neuropsychological test z‐scores. Between‐group differences in neurotransmitter‐informed FC were assessed using ANCOVA models in SMP12. Moreover, the association between FC maps and both Cog‐CS and episodic memory scores was investigated in the whole sample. Age, education, sex, total intracranial volume and recruitment site were included as covariates.

**Results:**

The AD group showed mesocorticolimbic DA‐related FC decline in the bilateral precuneus, and increase in the thalamus, while the MCI group showed nigrostriatal FC decline in the left superior temporal gyrus. ACh‐related FC decline was observed in both MCI and AD, compared with CU, in fronto‐parietal and temporal areas. The Cog‐CS was positively associated with ACh‐related FC only in posterior cingulate, temporal and cerebellar areas. Episodic memory performance was correlated positively with ACh‐ and DA‐related FC in occipito‐parietal areas and negatively with DA‐related FC in thalamic and fronto‐temporal areas.

**Conclusions:**

FC alterations associated with both Ach and DA may be sensitive to cognitive decline due to AD. These findings, first of this kind, may offer useful insights to test recently proposed hypotheses on the involvement of these neuromodulatory systems in driving AD‐related neuropathological changes as well as to investigate the mechanisms of action of novel treatments.

